# Adapting to the pandemic: longitudinal effects of social restrictions on time perception and boredom during the Covid-19 pandemic in Germany

**DOI:** 10.1038/s41598-022-05495-2

**Published:** 2022-02-03

**Authors:** Marlene Wessels, Nariman Utegaliyev, Christoph Bernhard, Robin Welsch, Daniel Oberfeld, Sven Thönes, Christoph von Castell

**Affiliations:** 1 Department of Experimental Psychology, Institute of Psychology, Johannes Gutenberg-University Mainz, Mainz, Germany; 2 Human-Centered Ubiquitous Computing, Ludwig-Maximilians-University München, Munich, Germany

**Keywords:** Psychology, Risk factors

## Abstract

With the Covid-19 pandemic, many governments introduced nationwide lockdowns that disrupted people’s daily routines and promoted social isolation. We applied a longitudinal online survey to investigate the mid-term effects of the mandated restrictions on the perceived passage of time (PPT) and boredom during and after a strict lockdown in Germany. One week after the beginning of the lockdown in March 2020, respondents reported a slower PPT and increased boredom compared to the pre-pandemic level. However, in the course of the lockdown, PPT accelerated and boredom decreased again until August 2020. Then, in October 2020, when incidence rates sharply rose and new restrictions were introduced, we again observed a slight trend toward a slowing of PPT and an increase of boredom. Our data also show that as the pandemic progressed, respondents adjusted their predictions about the pandemic’s duration substantially upward. In sum, our findings suggest that respondents adapted to the pandemic situation and anticipated it as the new “normal”. Furthermore, we determined perceived boredom and the general emotional state to be predictive of PPT, while depressive symptoms played a minor role.

## Introduction

The Covid-19 pandemic has mandated major changes and restrictions to our everyday life. To halt the transmission of the virus, the government of Germany initiated comprehensive measures that included a nationwide lockdown from March to May 2020. This lockdown disrupted people’s daily routines since it introduced self-isolation rules that restricted social contact and limited most leisure activities. Many workplaces and businesses were closed, threatening jobs and livelihoods, and whenever possible, people were encouraged to isolate themselves socially and work from home. Given the pervasive nature of the containment measures, the psychological and emotional impact of the Covid-19 pandemic has become a considerable research interest. In the present study, we examined how the perceived passage of time (PPT), the level of experienced boredom, indices of depressive symptoms, and the general emotional state changed in response to the restrictions adopted in Germany in the period from the introduction of the lockdown in spring 2020 to the subsequent easing in summer as well as to the second lockdown in fall 2020.

Early after the Covid-19 outbreak, the psychological impact of the pandemic was rated as moderate or severe in China^1^. Stricter and more far-reaching measures adopted in Hong Kong, as well as the home confinement rules introduced in France, were associated with high levels of perceived loneliness in addition to depression, anxiety, and stress^2,3^. During the summer of 2020 in South Korea, when the virus was temporarily contained but people were still advised to stay at home, respondents also indicated an increased level of anxiety, depression, stress, and loneliness^4^. Furthermore, the prolonged periods of reduced activities and social isolation increased boredom^5,6^, which constitutes an aversive psychological stressor^7^. According to Eastwood and colleagues^8^, boredom is defined as a negative state emerging from being unable to follow internal or external impulses necessary for participation in satisfactory events, while focusing on this inability and assuming external causes. In contrast to negative emotional valence, boredom is thus clearly associated with attentional processes. Taken together, people’s emotional state seems to be negatively affected by the social isolation measures during the Covid-19 pandemic, apparently independent of region and incidence rates. As emotional states are closely intertwined with perceptual and cognitive processes, it seems plausible to expect effects of social isolation on the latter two.

In particular, we expected the subjective perception of time to be impacted by the pandemic restrictions. It is a long-standing finding that negative affect, as well as boredom, are associated with a slowing of PPT^9,10^. More precisely, social isolation was associated with increased levels of boredom and negative affect over the course of the pandemic. Consistently, cross-sectional studies also observed a deceleration of the PPT during the Covid-19 pandemic^11^. With that in mind, the present study aimed to investigate the mid-term effects of boredom, depression, and the general emotional state on the PPT during and after a strict lockdown in Germany using a longitudinal survey method.

### Time perception

The perception of time is affected by various psychological factors, such as attentional focus and emotional states. The speed of the PPT—which we investigate in the present study—is typically measured with rating scales. For instance, participants are asked, “*How fast does time pass for you?*” and respond on a rating scale ranging from “*very slowly*” to “*very fast*”^12–14^. Only limited data are available on PPT, but there is converging evidence that PPT judgments are dissociated from duration judgments^13,15–18^.

PPT is tied to attention^19^, with participants typically reporting time to pass more quickly when engaged in a demanding task^20^ or when being in a state of mindfulness and meditation^21–23^. Both might divert attention from time, which is associated with an acceleration of PPT. In contrast, attention towards time, for example while waiting for a delayed bus, results in slower PPT^24^. Furthermore, decreasing arousal and increasing boredom, for example, when watching a movie repeatedly, were associated with slower PPT^9,24,25^. This indicates a link between the emotional state and PPT, which is in line with ecologically more valid assessments. Ratings of current PPT at various times of the day (but not duration judgments) were correlated with the emotional state, with the PPT experienced as faster when the arousal was higher^13^. In clinically significant depression, time is perceived to pass more slowly^16^, while in manic episodes, the subjective time seems to pass faster than in healthy controls^26^. As noted by Thönes and Oberfeld^16^, slower PPT in depression might be also mediated by boredom. Thus, attention towards the passage of time, boredom, and depressed mood are associated with a slower speed of PPT.

### Time perception during the Covid-19 pandemic

How do restrictions that limit social contact during the Covid-19 pandemic impact our PPT? The Covid-19 pandemic itself and the related social isolation measures present emotionally taxing challenges to many people and might enhance symptoms of depression or negative emotional states^27^. People spend more time at home during a lockdown, leading to repetitive routines lacking diversified events, which, in turn, promotes an unpleasant state of boredom^7,28^.

There is first evidence for an effect of the pandemic restrictions on the subjective perception of time from studies that were conducted in parallel with our data collection. In two cross-sectional studies investigating PPT during a period of the first lockdown in the UK in April 2020^29^ and the second lockdown in England^30^, the respondents were asked to rate PPT both for the current day and the current week as well as their emotional state, task load, and satisfaction with social interactions. The majority of the respondents of both studies reported PPT to be distorted during the lockdown compared to before. Although some respondents indicated an acceleration and others a slowing down of PPT, the latter was associated with higher levels of stress and depression, as well as with a reduced level of satisfaction and task load.

A distinct effect of pandemic restrictions on PPT was indicated in a cross-sectional study in Italy, conducted two weeks after the introduction of a nationwide lockdown in March 2020^31^. They administered an online survey including items regarding sleep quality, digital media use, subjective experience of time, and emotional state. Comparing the lockdown to the first week of February 2020 (when no restrictions were imposed), respondents reported the time to pass more slowly, the feeling of being “stuck in time” more frequently, and an increased feeling of boredom. One-fourth of the respondents also indicated increased symptoms of depression and/or anxiety, while half of the respondents reported moderate to severe symptoms of stress and/or poor sleep quality. Although boredom and PPT ratings were reported in this study, no direct relationship was established between these two measures.

The interplay of PPT and boredom with respect to emotional experiences during pandemic-related restrictions has been more closely examined in two longitudinal studies in France^32^. Respondents reported a slower PPT during the first lockdown (in April 2020) as compared to before the lockdown, which did not recover to a “normal” level neither after six months, nor after one year. The slowed PPT was primarily associated with an increased feeling of boredom.

Taken together, previous studies indicate an effect of social isolation on the emotional state, boredom, and PPT. However, previous studies were either cross-sectional or captured measurement points with a large time interval of half a year. Hence, no conclusions regarding flexible changes and/or longitudinal adaptation of PPT in response to current restrictions can be drawn from the previous data. To investigate this, it is essential to capture a more precise time course of PPT, boredom, and emotional state in periods of social isolation during a lockdown, but also when the restrictions are relaxed again.

From a theoretical point of view, the PPT judgments being based on “self-duration”^19^ reflect the results of self-observation in a given situation. During the persistent period of home confinement, people may first feel the time as dragging, but after a while cope with the new “normal”. That being said, we expect such adaptational processes to be reflected in the PPT judgments. To capture the evolution of coping strategies, it is necessary to assess repeated PPT judgements within the “same” situation. With a changing situation where some lockdown rules are lifted or new are announced, people may benefit from their improved strategies and transfer them to the changed pandemic situation.

We conducted an online survey at 10 closely spaced measurement points during spring 2020. The survey was minimized to items relevant for the preregistered hypotheses to minimize the participation threshold and to increase the level of respondent retention. We expected the PPT to slow down, boredom to increase, and the emotional state to deteriorate during the course of a German-wide lockdown from March to May 2020. In accordance with previous research, we expected a slower PPT to be associated with increased boredom and a deteriorated emotional state. We supplemented this survey period with two follow-up measurements, one in mid-August, and the other in late October, to additionally explore changes and potentially adaptive processes in these outcomes in periods of relaxed regulations.

## Method

### Respondents

Respondents were recruited via local mailing lists and social networks. Only respondents who completed the survey during the first measurement point were included in the sample since it contained questions on demographic information (*n*_*MP1*_ = 188). The 136 respondents who completed at least 7 of the 10 measurement points of the survey were included in the final analyses. Exceeding the preregistered minimum of 50 respondents (As Predicted #38026), the sample (114 female, 22 male, age: *M* = 29.40 years, *SD* = 11.75 years) was composed of 72.8% students, 23.5% workers, 2.2% retired and 2.2% unemployed. All respondents stated to be of legal age (≥ 18 years), and to be residing in Germany. The residence was restricted to Germany to ensure similar pandemic-related restrictions and events for all respondents. The study was conducted in accordance with the ethical principles of the Helsinki Declaration and approved by the Ethics Committee of the Institute of Psychology of the Johannes Gutenberg-University Mainz (2020-JGU-psychEK-S003). All respondents provided written informed consent at the beginning of each survey, participated on a voluntary basis or, in case of students, for partial course credit, and could leave questions unanswered.

### Survey

The survey was released online via SoSci Survey (version 3.2.00) on 30th March 2020, ended on 1st May 2020, and included 10 measurement points (main study). For the first measurement point, we accepted a response period of 4 days, since it additionally included relevant demographic questions. The responses on the following 9 measurement points were collected on the same day the survey was released. Two additional measurement points on 14th/15th August 2020 (*n* = 66; 56 female, 10 male; age: *M* = 31.35 years, *SD* = 12.13 years) and 30th/31st October 2020 (*n* = 52; 43 female, 9 male; age: *M* = 29.83 years, *SD* = 11.95 years) were offered to provide a deeper understanding of longitudinal effects in the unexpectedly prolonged pandemic (follow-up study).

The survey took approximately 5–10 min to complete and contained 14 items regarding time perception, boredom, emotional state, depressive symptoms, and interpersonal distance preferences. In this article, we focus on the first four measures, but findings on interpersonal distance preferences are reported in Welsch et al.^33^. Two items measured PPT during the last three days (*pandemic PPT;* “How quickly did time pass for you within the last three days?”) and before the pandemic (*pre-pandemic PPT;* “How quickly did time pass for you under pre-pandemic circumstances, i.e., before the Corona crisis?”). Note that the repeated measurement of *pre-pandemic* PPT enabled us to assess a potential hindsight bias regarding the retrospective judgments. We additionally asked respondents to estimate in weeks how long they thought the pandemic would last (“For how long do you think the Corona crisis will still last?”). The two items of boredom (*pandemic* and *pre-pandemic boredom*) were probed as follows: “How bored did you feel within the last three days/under pre-pandemic circumstances, e.g., before the Corona crisis?” Both PPT and boredom were measured on 7-point rating scales ranging from “very slow”/“not bored” to “very fast”/“very bored”, respectively. To assess the individual emotional state within the last three days, we used the non-verbal five-point Self-Assessment Manikin (SAM) scale for valence^34^ as well as the two-item Patient Health Questionnaire (PHQ-2) for detecting depressive symptoms^35^. For the SAM scale, values higher than three were regarded as a positive emotional state, while values lower than 3 indicated a negative emotional state; a value of 3 represented a “neutral” state. The PHQ score can range between 0 and 6, with higher scores indicating more depressive symptoms.

### Follow-up study

To further explore longitudinal effects in the ongoing pandemic, we collected data on two additional measurement points, which were timed 15 weeks (measurement point 11, August 2020) and 26 weeks (measurement point 12, October 2020) after the period of the main study had finished. In the follow-up analyses, we compared measurement points 10 and 11, and measurement points 11 and 12, respectively. Note that only subjects who participated in both measurement points were included in the respective analysis (measurement points 10 vs. 11: *n* = 64, 55 females, 9 males, age: *M* = 31.59 years, *SD* = 12.24 years; measurement points 11 vs. 12: *n* = 33, 30 females, 3 males, age: *M* = 30.94 years, *SD* = 12.76 years).

### Pandemic regulations in Germany

In order to give a deeper insight into the pandemic situation in Germany during the survey period, we list the national pandemic regulations^36^ and Covid-19 incidences in Table 1.

**Table 1:** Pandemic regulations in Germany during the survey period, adapted from Imöhl and Ivanov^36^.

Measurement point	Date (2020)	Covid-19 incidences	German pandemic regulations
	19th March	+ 2801	Closing of schools and kindergartens
	22nd March	+ 1948	Closing of restaurants, stores, museums, meeting of max. 2 persons in public (rules planned for two weeks)
1	30th March	+ 4751	
2	3rd April	+ 6174	
3	6th April	+ 3677	14 days quarantine obligation after travel return from other countries
4	10th April	+ 5323	RKI announced worldwide spreading of the virus
5	13th April	+ 2,537	Rules for commuters and travelers
	15th April	+ 2486	Restricted contact rules and school/kindergarten closings prolonged for three weeks
6	17th April	+ 3380	Decision to gradually lift lockdown rules
7	20th April	+ 1775	Opening of stores and schools for graduating classes, mandatory mask-wearing in single federal states
8	24th April	+ 2337	European credit aids passed
9	27th April	+ 1018	Mandatory mask-wearing in whole Germany
10	1st May	+ 1639	Opening of playgrounds, museums, parks, first human vaccine studies
	15th June	+ 192	Return to “normal” public life
11	14th August	+ 1449	14 days quarantine obligation or negative Covid-19 test after travel return from risk areas
12	30th October	+ 18681	Announcement of the second nation-wide lockdown

The regulations came into force either on the measurement point/date indicated or in the period between two measurement points/dates. The incidences were published by the Robert Koch Institute (RKI) at the given measurement point/date.

## Results

Figure ﻿1 shows the mean *pandemic* and *pre-pandemic* PPT and boredom ratings throughout the survey period. We first refer to the data collected in the main study and then integrate the data from the two follow-up measurements. We calculated two linear mixed-effects models to examine the *pandemic* PPT and boredom ratings throughout the course of the restrictions from March until April 2020 (main study) in more detail. All statistical analyses were performed using R Studio (version: 1.3.959, R version: 3.6.3, R Core Team^37^). The models were created with the lme()-function from the nlme R package^38^ and used the restricted maximum-likelihood method for estimation of the variance components. We included the difference-coded categorical predictor *measurement point* (i.e., differences between the second and first, the third and second measurement point, and so on) as a fixed effect to determine the influence of the time course, and the *respondent* as a random intercept to account for interindividual differences and the repeated measures structure of our data. All results were interpreted on a significance level of α = 0.05. With its random and fixed effects, the model predicting *pandemic* PPT explained 55.65% of the variance, and the model predicting *pandemic* boredom explained 61.98% as obtained using a measure of *R*^2^_*conditional*_^39^. Two Type-3 *F-*tests performed on the parameters of these models confirmed that *pandemic* PPT, *F*(9,1128) = 5.57, *p* < 0.001, and *pandemic* boredom, *F*(9,1128) = 18.94, *p* < 0.001, varied significantly between measurement points. As revealed by the significant positive model estimates in Table 2, the *pandemic* PPT substantially increased between the 1st and 2nd as well as between the 5th and 6th measurement point. For the *pandemic* boredom, the significant negative model estimates (Table S1, Supplement) indicated that the perceived boredom substantially decreased between the 1st and 2nd, 3rd and 4th, and 7th and 8th measurement point. These differences reveal longitudinal effects of the pandemic-related events with a trend towards an accelerating PPT and a reduction of boredom during the pandemic.

**Figure 1:**
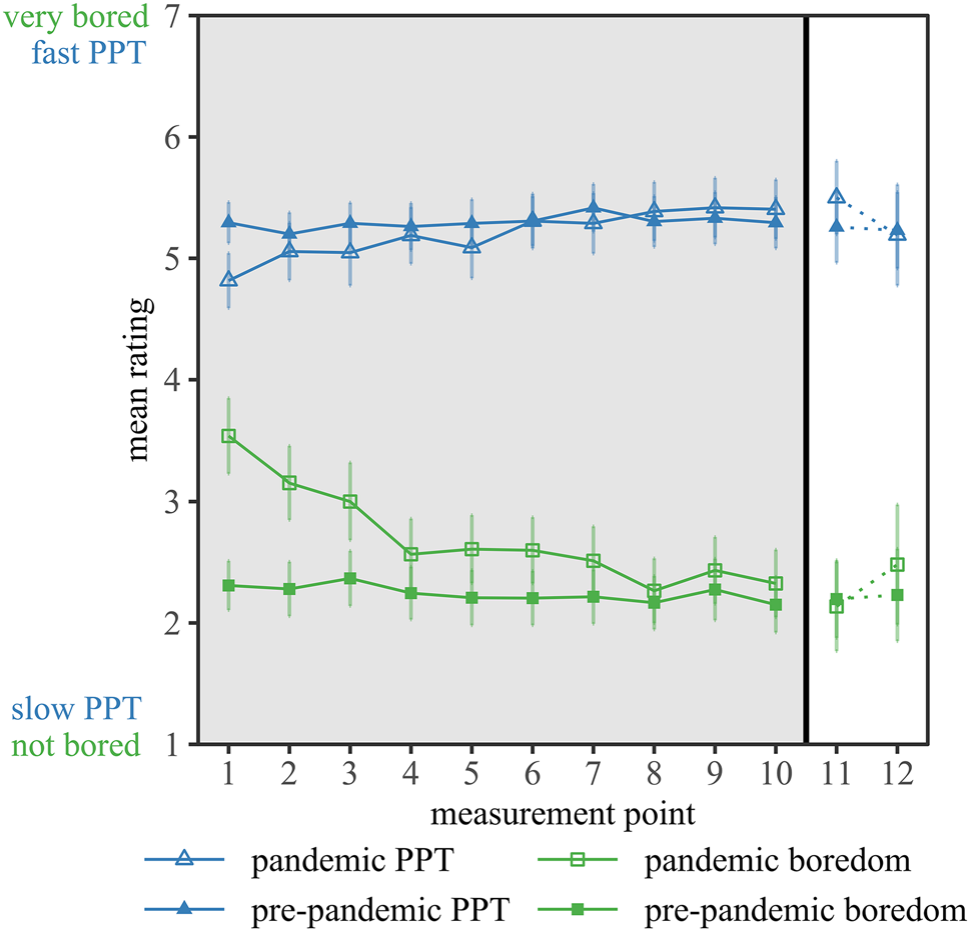
Mean ratings for the perceived passage of time (PPT) and boredom, both before (*pre-pandemic*) and during the pandemic as a function of measurement points. The main study included measurement points 1–10 (gray background). The follow-up study included measurement points 11 and 12 (white background). Error bars show 95% confidence intervals.

**Table 2:** Estimated fixed effects parameters of the model predicting pandemic PPT from the measurement point (MP).

	*β*	*SE*	*df*	*t*	*p*
(Intercept)	5.21	0.09	1128	56.87	< 0.0001
MP 2–1	0.24	0.12	1128	2.12	0.0343
MP 3–2	0.03	0.12	1128	0.27	0.7907
MP 4–3	0.14	0.12	1128	1.16	0.2464
MP 5–4	− 0.13	0.12	1128	− 1.11	0.2687
MP 6–5	0.23	0.12	1128	1.97	0.0497
MP 7–6	− 0.06	0.12	1128	− 0.53	0.5990
MP 8–7	0.12	0.12	1128	1.03	0.3041
MP 9–8	0.03	0.12	1128	0.23	0.8159
MP 10–9	− 0.01	0.12	1128	− 0.09	0.9244

Shown are effect estimates (*β*), standard errors (SE), degrees of freedom (df), t and p values. The difference coding compares the means of two consecutive measurement points.

**Figure 2:**
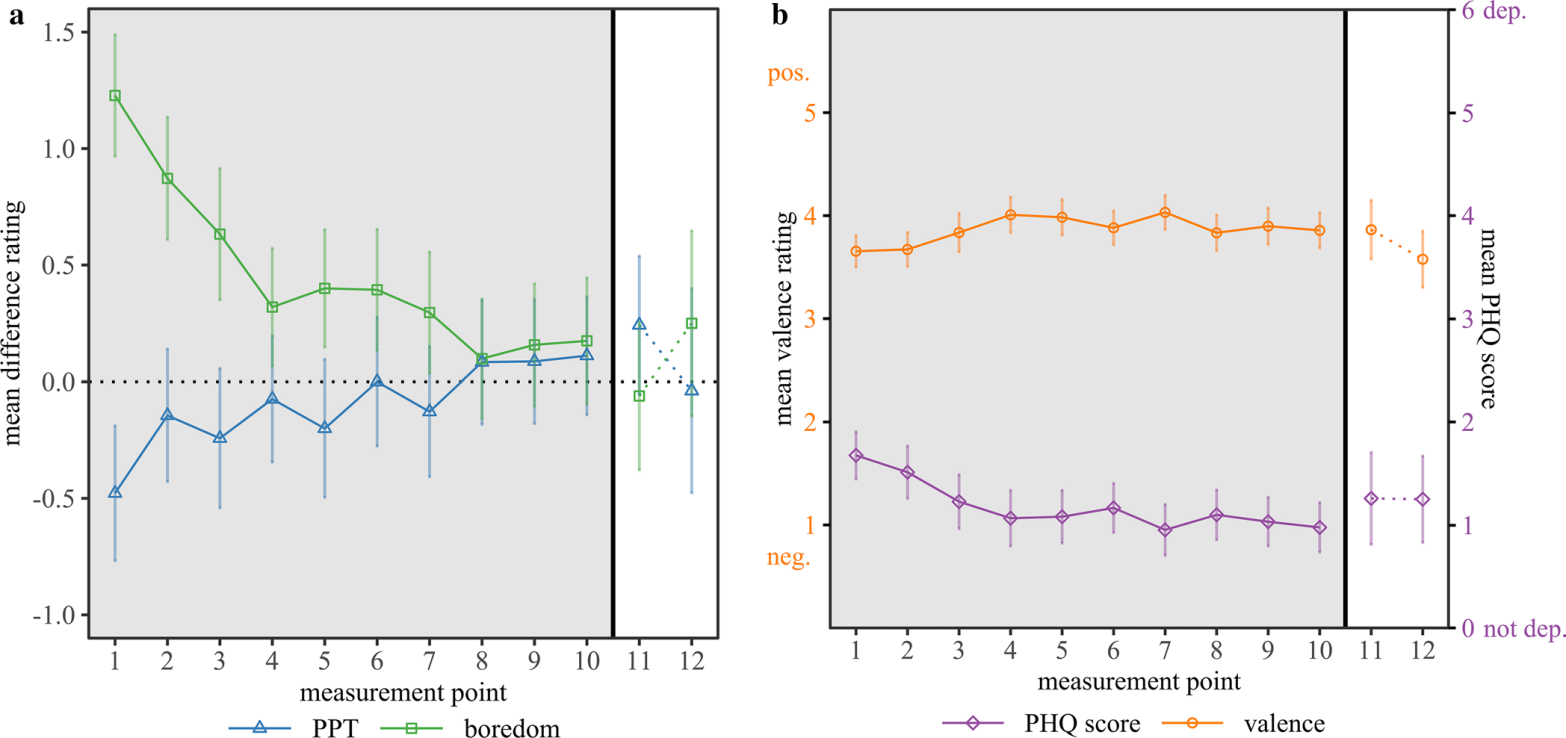
(**a**) Mean differences between the *pandemic* and *pre-pandemic* ratings are depicted for the perceived passage of time (PPT) (blue triangle) and boredom (green square), as a function of measurement point. The dotted horizontal line marks the level of no difference between *pandemic* and *pre-pandemic* ratings. Positive values indicate faster *pandemic* PPT/higher pandemic boredom compared to the *pre-pandemic* ratings. (**b**) Mean valence ratings (left y-axis) and mean PHQ scores (right y-axis) during the pandemic are displayed as a function of measurement point. Note that higher PHQ scores (purple diamond) indicate more depressive symptoms. Higher valence ratings (orange circles) indicate a positive emotional state, while lower values represent a negative emotional state. A value of 3 indicates a “neutral” state. The main study included measurement points 1–10 (gray background). The follow-up study included the measurement points 11 and 12 (white background). Error bars show 95% confidence intervals of the mean.

Using the same statistical approach as for the *pandemic* ratings, the Type-3 *F-*tests indicated that *pre-pandemic* PPT, *F*(9,1128) = 0.61, *p* = 0.791, *R*^2^ = 0.71, and *pre-pandemic* boredom, *F*(9,1128) = 1.53, *p* = 0.133, *R*^2^ = 0.75, remained largely stable between the measurement points, which indicates that the retrospective measurement of* pre-pandemic *PPT was largely unaffected by a hindsight bias. To visualize the direct relation between *pandemic* and *pre-pandemic* PPT and boredom ratings, we first calculated the difference between *pandemic* and *pre-pandemic* ratings per respondent and measurement point. Figure 2a depicts the mean differences as a function of measurement point. Rating differences for PPT and boredom both converged to a level near zero towards the end of the main study period. Given the largely stable *pre-pandemic* ratings, this longitudinal trend can be attributed to the acceleration of the *pandemic* PPT and the reduction of the *pandemic* boredom towards *pre-pandemic* levels.

Figure 2b displays a trend towards increasing valence ratings and decreasing PHQ scores (reduced symptom burden) across the measurement points, indicating an improvement of the emotional state and a reduction of depressive symptoms across the main study period. To statistically examine the factors contributing to the acceleration of the *pandemic* PPT, we initially inspected the mean correlation coefficients between the *pandemic* PPT, boredom, valence and PHQ scores and applied a stepwise regression approach. The mean correlations were calculated as correlations per measurement point and subsequently averaged across measurement points. Based on the correlation strength with *pandemic* PPT, we first added the predictor *pandemic* boredom (model 2), *r*_*mean*_ = − 0.50, *CI*_*95%*_ = [− 0.56, − 0.44], to the aforementioned model predicting the *pandemic* PPT from the predictor measurement point (model 1). In a second step, we added the predictor valence, *r*_*mean*_ = 0.32, *CI*_*95%*_ = [0.29, 0.36] to the model (model 3). The predictor PHQ score was added last (model 4), *r*_*mean*_ = − 0.30, *CI*_*95%*_ = [− 0.34, − 0.25].

We compared the four models resulting from the stepwise regression using Log-Likelihood Ratio tests, Akaike information criteria (AIC), Bayesian information criteria (BIC), and *R*^2^ (see Table 3). Note that for this model comparison, we used a maximum-likelihood method for estimation of the variance components. Adding *pandemic* boredom ratings to the model (model 2, see also Table S2, Supplement) improved the model prediction compared to model 1. When subsequently adding the valence ratings as a predictor (model 3), the model prediction improved significantly further. Thus, the emotional state was substantially associated with the PPT during the pandemic and explained aspects of the PPT not yet explained by the time passed since the beginning of the lockdown and boredom. In contrast, adding the PHQ score as an additional predictor to the model did not significantly contribute to the prediction of *pandemic* PPT. As the PHQ score was highly correlated with the valence ratings, *r*_*mean*_ = − 0.68, *CI*_*95%*_ = [− 0.71, − 0.64], it may be assumed that the variance explained by depressive symptoms and the emotional state had a great overlap. This may be one possible reason for the lacking incremental improvement of the model prediction by including the PHQ as a predictor (alternative explanations are outlined in the discussion section). Taken together, *pandemic* PPT was best and most parsimoniously predicted by the measurement point, boredom, and valence ratings. Thus, the observed longitudinal acceleration of PPT was closely associated with decreasing levels of boredom and an improvement in the general emotional state throughout the mid-term course of the pandemic.

**Table 3:** Stepwise regression. In a stepwise manner, the *pandemic* boredom ratings, valence ratings and PHQ scores are added to the model predicting the *pandemic* PPT from the measurement points (MP) of the main study.

Model	Predictors		*df*		*AIC*		*BIC*		*R*^2^		*LL*		*Test*		*L. *ratio		*p*	
1	MP		12		3808		3870		0.56		− 1892.20							
2	MP + boredom		13		3607		3674		0.59		− 1790.70		1 vs. 2		203.01		< 0.001	
3	MP + boredom + valence		14		3589		3661		0.59		− 1780.52		2 vs. 3		20.35		< 0.001	
4	MP + boredom + valence + PHQ		15		3594		3671		0.59		− 1781.96		3 vs. 4		2.87		0.090	

For the statistical model comparisons, the degrees of freedom (df), Akaike information criteria (AIC) and Bayesian information criteria (BIC), R^2^, Log-Likelihood (LL), log-likelihood ratio (L. Ratio) and p-values are reported.

The negative estimate of boredom and the positive estimate of valence in model 3 (Table 4) indicate that high levels of *pandemic* PPT were associated with low levels of boredom and high levels of valence. In other words, respondents being less bored and/or in a better emotional state were more likely to perceive the time during the pandemic to pass faster than respondents being more bored and/or in a worse emotional state.

**Table 4:** Estimated fixed-effects parameters of the regression model (model 3) best and most economically predicting the *pandemic* PPT.

	*ß*	*SE*	*df*	*t*	*p*
(Intercept)	5.32	0.19	1126	28.02	< 0.001
MP 2–1	0.12	0.11	1126	1.11	0.265
MP 3–2	− 0.05	0.11	1126	− 0.49	0.621
MP 4–3	0.00	0.11	1126	− 0.01	0.989
MP 5–4	− 0.13	0.11	1126	− 1.23	0.221
MP 6–5	0.24	0.11	1126	2.19	0.029
MP 7–6	− 0.09	0.11	1126	− 0.79	0.430
MP 8–7	0.06	0.11	1126	0.55	0.580
MP 9–8	0.07	0.11	1126	0.65	0.514
MP 10–9	− 0.04	0.11	1126	− 0.35	0.728
Boredom	− 0.31	0.02	1126	− 12.93	< 0.001
Valence	0.19	0.04	1126	5.04	< 0.001

The model included the predictors measurement point (MP), boredom and valence. Shown are effect estimates (*β*), standard errors (SE), degrees of freedom (df), t and p values. The difference coding compares the means of two consecutive measurement points.

### Follow-up study

Because the number of respondents decreased between the main and follow-up study, we focus on descriptive statistics to assess the plausibility of the model predictions evolved from the main study. Still, we include two-sided paired-samples *t*-tests and report Cohen's *d*_*z*_ as a sample-size independent measure to examine the differences between measurement points in the follow-up study. Note that the samples underlying these tests were reduced to those respondents who participated in both measurement points being compared (*n* = 64 respondents for the comparison of measurement points 10 and 11, and *n* = 33 respondents for measurement points 11 and 12).

At measurement point 11, the respondents reported PPT as slightly faster, *d*_*z*_ = 0.16, and to feel a little less bored, *d*_*z*_ = 0.06, compared to the last measurement point of the main study (Fig. 1). At measurement point 12, the respondents judged *pandemic* PPT to be slower, *d*_*z*_ = 0.11, and *pandemic* boredom to increase again, *d*_*z*_ = 0.09. However, these descriptive differences are fairly small and failed to reach significance, all *p* > 0.214. Hence, our data provide only a first descriptive indication for a dynamic and bidirectional adaptation of the respondents’ PPT and boredom in response to the measures enacted during the pandemic.

The low variability of the *pre-pandemic* ratings during the main study was further supported by the follow-up data. Even though the intervals between the main study and the follow-ups comprised 15 and 26 weeks, respectively, the retrospective judgments of the *pre-pandemic* PPT and boredom were descriptively at level with the main study. Taken together, respondents reported largely stable retrospective judgments of a relatively fast PPT and relatively low levels of boredom for the period prior to the pandemic, even after a substantially long period of time.

The aforementioned association between the subjective *pandemic* PPT and the general emotional state, as operationalized by the valence ratings, was additionally observed during the follow-up period. In line with the regression model for the *pandemic* PPT based on the data of the main study, respondents indicated a good emotional condition when time was perceived as passing rather quickly (measurement point 11), while they reported a deteriorated emotional state when the PPT slowed again (measurement point 12). In sum, the descriptive data of the follow-up study were in line with the effects observed in the main study, and they further suggest a bidirectional dynamic between the PPT and boredom with (the prospect of) the social isolating regulations and/or the general emotional state during the pandemic. We elaborate on the potential implications of this additional finding in the discussion section.

### Estimated duration of the pandemic

**Figure 3:**
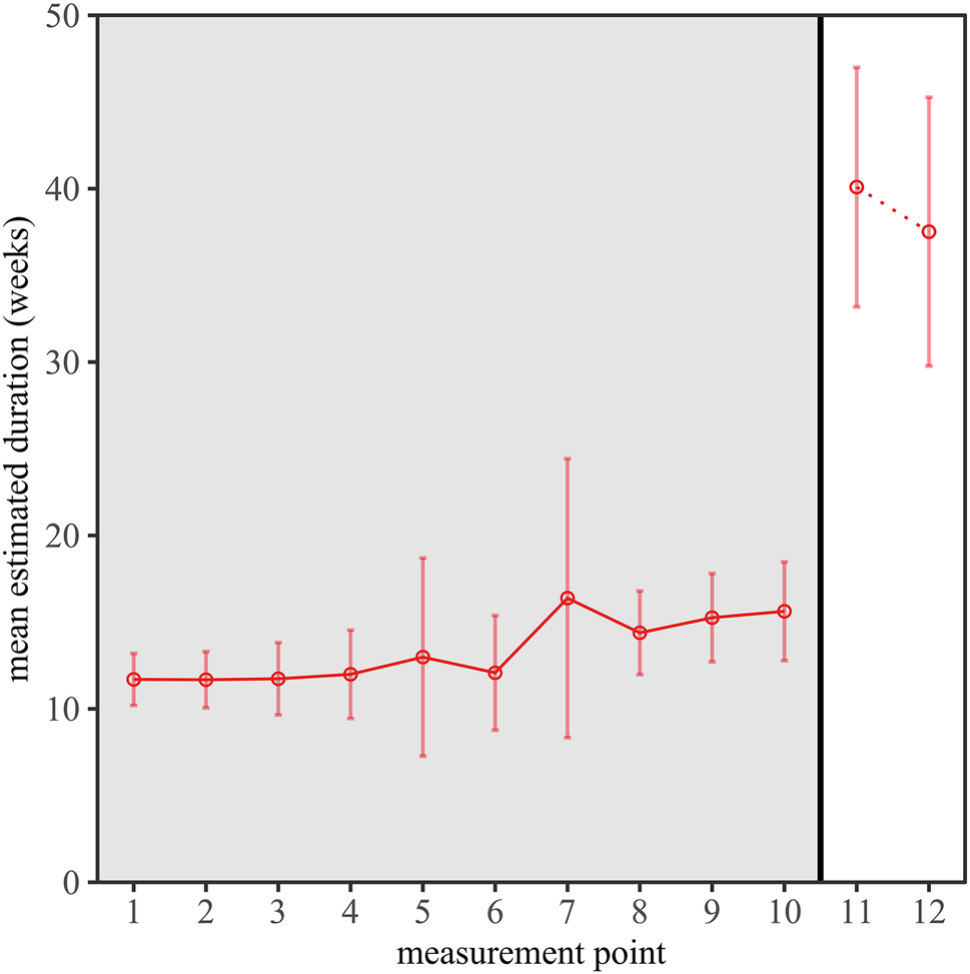
Mean estimated durations of how long respondents expected the pandemic to last as a function of measurement point. The main study included measurement points 1–10 (gray background). The follow-up study included measurement points 11 and 12 (white background). Error bars show 95% confidence intervals.

Additionally, the respondents were asked how long they thought the pandemic would last. This prediction provided further insight into the respondents’ mindsets, i.e., what they expected to happen in the future. Figure 3 shows the estimated duration of the pandemic as a function of the measurement point. At the beginning of the survey period, respondents estimated the pandemic to last approximately 11 weeks on average. Throughout the main study, estimates fluctuated to some extent but tended to increase over time. At the end of the main study, respondents expected the pandemic to continue for another 15 weeks on average. In an explorative analysis, we compared the duration predictions at measurement point 1 and 10 and calculated a paired-samples *t*-test (two-tailed) for the subsample of respondents who participated in both measurement points (*n* = 126). This comparison was significant, *t*(125) = − 3.66, *p* < 0.001, *d*_*z*_ = − 0.33. Hence, respondents substantially adjusted their duration predictions between the beginning and the end of the main study in spring 2020. However, the correlations between the expected duration of the pandemic and *pandemic* PPT, *r* = 0.053, and *pandemic* boredom, *r* = 0.004, were very small. Thus, the expected duration of the restrictions appears to have little to do with PPT and the feeling of boredom during the pandemic.

In the follow-up study in August 2020 (see again Fig. 3), we found a strong increase of the mean duration predictions (*M* = 40.09 weeks, *SD* = 1.23 weeks) relative to the period of the main study. A *t*-test comparing the predicted durations at measurement points 10 and 11 showed that respondents once again significantly increased their duration predictions, *t*(63) = − 6.26, *p* < 0.001, *d*_*z*_ = − 0.78. At measurement point 12, we see a slight decrease in the estimated durations (*M* = 37.52 weeks, *SD* = 1.50 weeks) compared to measurement point 11, although not statistically significant, *t*(32) = 0.70, *p* = 0.491. Taken together, respondents expected the pandemic to last increasingly longer between spring and summer 2020, with their estimate remaining roughly level between summer and fall 2020.

## Discussion

We used a longitudinal design to investigate the mid-term effects of reduced social contact on the perceived passage of time (PPT) and boredom during and after a strict lockdown in Germany. One week after the beginning of the lockdown, respondents reported a slower PPT and increased boredom compared to pre-pandemic levels. During the subsequent measurements, as incidence rates decreased and the socially isolating restrictions were gradually relaxed, respondents’ PPT ratings accelerated again, and levels of boredom decreased. In the follow-up study, when the incidence sharply rose again, and a second lockdown was announced by the government, PPT decreased and boredom increased again. We further determined factors that predicted *pandemic* PPT. That is, the time course of the pandemic (as operationalized by the measurement point), perceived boredom, and the general emotional state were indicative of PPT. Depressive symptoms, in contrast, played a minor role. In sum, our results suggest an adaptive nature of PPT during the pandemic. The analysis of the duration predictions throughout the survey period additionally indicated that respondents flexibly updated their expectancies regarding the expected persistence of the pandemic.

In our longitudinal study, the respondents first indicated a considerably slowed down PPT and increased levels of boredom than before the pandemic. This is compatible with the results of two cross-sectional studies that were conducted two weeks after the beginning of the first lockdown in France^11,40^. At the second measurement point of our survey, however, the respondents indicated a progressive acceleration of the PPT and decreased levels of boredom as compared to the first measurement point. Although the reported PPT and boredom still differed from the retrospective ratings for the time prior to the pandemic, this prompts an adaptive nature of *pandemic* PPT and boredom. It appears that respondents had begun to adapt to the new constraints and, thus, that the initial picture of a decelerated *pandemic* PPT and increased boredom would not be a permanent condition. As the study progressed, we further observed that the *pandemic* PPT and boredom ratings converged more and more with the *pre-pandemic* ratings, compatible with a progressive adaptation.

As outlined by Wittmann^28^, more memorable events are formed in a variably changing rich environment compared to an environment characterized by routine and monotony. When recalling the abundance of numerous events from a period of time, in retrospection, it feels as if this period had a rather long duration because “so much happened”. In contrast, a period during the lockdown may have held less memorable and more repetitive events that, in retrospection, might have given the impression that this period was comparably short because “nothing happened”. On this basis, Wittmann^28^ suggested that the PPT should rather accelerate than decelerate during the pandemic. However, our data is clearly in favor of a deceleration of the *pandemic* PPT as compared to *pre-*pandemic levels. This underlines the conceptual difference (see “Introduction”) between retrospective duration judgments, as discussed by Wittmann^28^, and retrospective PPT ratings, as assessed in the present study. While the judgment of a duration of a past period in time is supposed to be predominantly influenced by the availability of memorized events, the PPT ratings in the present study were strongly related to the respondents’ internal states of boredom and valence. In the present study, we observed that the *pandemic* PPT accelerated even before restrictions were lifted. This cannot be explained by changes in the external environment but is compatible with a substantial influence of internal processes on PPT during the pandemic. It prompts the assumption of a progressive internal adaptation of PPT.

In this context, the duration predictions gave further insight into what respondents expected to happen in the future. In our exploratory analyses, we observed that respondents increased their predictions of the remaining duration of the pandemic throughout the main study and most strongly between the main and the follow-up period. Be reminded that at the beginning of the main study, our respondents predicted the pandemic to last for another 11 weeks on average. In the follow-up period, these predictions increased to an average duration judgement of 40 weeks at measurement point 11. This suggests flexible adaptation of the predictions to the available level of information and might reflect that respondents also anticipated this exceptional situation to become the new “normal”. Although the expected duration of the pandemic appears to have little to do with PPT and boredom, it could still support the assumption of progressive internal adjustment processes.

With this in mind, the mid-term acceleration of the PPT during the lockdown period could be related to coping strategies. In fact, Witowska et al.^24^ demonstrated that better self-regulation in everyday life was associated with a reduced feeling of boredom and a faster PPT during a waiting situation. Although they focused on interindividual differences in their cross-sectional study, it seems highly plausible that one can also improve the individual self-regulation over time. Shortly after the pandemic measures were introduced, the respondents were challenged to deal with the novel situation and might have had trouble distracting themselves, and thus felt increasingly bored and experienced the time to drag. However, it seems as if our respondents quickly developed adequate coping strategies that resulted in a reduced feeling of boredom and acceleration of PPT. This assumption is compatible with a longitudinal study conducted in the UK, demonstrating a steep increase of psychological distress during the initial pandemic period and a gradual decrease in the preceding pandemic^41^. Taken together, the progressive adaptation of boredom and PPT during the pandemic might be due to the fast development and improvement of efficient coping strategies to temporarily deal with the social isolation.

Particularly between measurement points 8 to 10, we observed mean *pandemic* PPT and boredom ratings almost at level with *pre-pandemic* ratings—shortly after the government had relaxed the restrictions and partially reopened stores, museums, and schools again (around measurement point 7). This highlights the sensitivity to external events and to the prospect of regained social activities, although adequate coping strategies might have been developed. It is not surprising that the regained leisure or employment opportunities were closely related to decreased levels of boredom. But the relaxation of the restrictive measures also had an effect on the cognitive level in terms of an accelerated *pandemic* PPT. Therefore, our longitudinal data suggest adaptive processes underlying PPT and boredom during the lockdown and emphasize also their sensitivity to pandemic-related events.

Although we refrained from including the data collected during the follow-up study in the main analyses due to the strongly increased drop-out rate, the data were descriptively in line with the adaptive nature of PPT and boredom. In summer 2020 (measurement point 11) when the regulations were relaxed, the respondents felt just as bored as before the pandemic and perceived the time to pass slightly faster. In fall 2020 (measurement point 12), when the second lockdown was announced and imminent, boredom slightly increased again and PPT slowed to the *pre-pandemic* level. This is compatible with two longitudinal studies by Droit-Volet et al.^32^, who found that respondents perceived the passage of time as slowed during pandemic restrictions in France in April 2020 as well as in November 2020, and even April 2021. These studies might reflect the sensitivity to pandemic-related events rather than an internal adjustment process, as the measurement points were at a much coarser time scale than in our study, but most importantly they were all during a lockdown period. As noted above, our follow-up findings should be interpreted with some caution; however, they may suggest that people were responding to current pandemic-related events, but to a presumably lesser extent than observed in the first phase of the main study. If these findings could be confirmed in a future study, it would suppose a bidirectional dynamic adaptation of PPT and boredom to both social restrictions and relaxations, highlighting the potential reversibility of the deteriorating effect of the pandemic.

Moreover, the dynamic interplay of boredom and PPT previously found in cross-sectional studies^40^ finds support in our longitudinal data. Over the course of our main study, an accelerating *pandemic* PPT was associated with a reduction of boredom as well as with an improvement of the general emotional state. This longitudinal dynamic is again compatible with the US study by Daly and Robinson^42^ demonstrating a gradual reduction of psychological distress, which they discuss in light of resilience and an effective adaptation to the pandemic.

The latter finding might imply that depressive symptoms also played an important role in predicting *pandemic* PPT in the present study (see also Thönes and Oberfeld^16^). However, our data could not confirm this assumption as PHQ score failed to significantly predict *pandemic* PPT. This could be for three reasons. First, the modification of the PHQ items might have reduced their reliability and/or validity. As outlined in the methods section, we set the time period for which the frequency of depressive symptoms was to be indicated to the last three days (instead of the last 14 days). This modification was necessary due to the closely timed measurement points. Second, our respondents generally indicated only marginal depressive symptomatology, as confirmed by consistently low PHQ scores. This is in accordance with another longitudinal study reporting only a slight increase of depressive symptoms in a large sample of university students^43^. Also, a meta-analysis indicated only a weak change in the prevalence of anxiety in students prior and during the pandemic^44^. With this in mind, the restricted variance in our predominantly student, healthy sample might have prevented the PHQ from predicting incremental variance of the *pandemic* PPT in addition to the general emotional state, as operationalized by the valence ratings. Third, the meta-analysis by Bueno-Notivol and colleagues^45^ pointed out that the different prevalence of depression during the Covid-19 pandemic reported can be partly attributed to different measures being used. A lower prevalence was indicated with the PHQ-9 compared to the Depression, Anxiety and Stress Scale (DASS). Thus, the use of the PHQ-2 in the present study, a short version of the PHQ-9, might have favored comparatively low estimates of depressive symptomatology. This could also explain why studies using the DASS reported a higher psychological burden of the pandemic^1^. For instance, Mazza et al.^46^ measured depressive symptoms using the DASS and reported that of 2766 Italian respondents 17% had high and 15.4% had extremely high levels of depression. Wang et al.^1^ also used the DASS and found that 16.5% of 1210 Chinese respondents reported moderate to severe depressive symptomatology.

In sum, the general emotional state of the respondents was predictive of the PPT during the pandemic, whilst clinical depressive symptomatology was not. Nevertheless, we emphasize that the role of depression for PPT in the present study may have been underestimated for the reasons mentioned before.

### Generalizability of the present findings

Our longitudinal study comprised a relatively large sample for the main survey period promoting the statistical power of our findings. Nevertheless, the results may not be fully representative of the general population, as the sample predominantly included female university students and middle-aged people. This might have also contributed to the aforementioned low variability of the depressive symptomatology. Assessing responses of a more heterogeneous sample might have led to more variation in clinical depressive symptomatology.

Moreover, there was a substantial drop-out between the main and the follow-up study. For the follow-up measurement points 11 and 12, we had *n* = 66 and *n* = 52 respondents, respectively, which was still above the preregistered minimum of *n* = 50. We decided to separate the results of the main and follow-up study for analyses and focus on descriptive patterns in the follow-up data. Here, we stress not to overestimate these descriptive patterns, but to consider them as initial validation of the regression model constructed from the data of the main study. Future research is needed to validate the role of specific events, boredom and the general emotional state with regard to PPT.

Previous studies also indicated that experienced sleep quality^31^, life rhythm^32^, anxiety and stress^29–31^ were associated with PPT and boredom during the pandemic. Our main interest was to examine the mid-term effects of specific events, boredom, and emotional state on PPT. Therefore, we limited the survey to the most important items with regards to our research questions in order to maintain motivation to voluntarily participate in our study on a regular basis over a quite extended period of time. Future studies should also include other factors to deepen the understanding between PPT and e.g. sleep or anxiety disorders.

Lastly, we demonstrated that retrospectively assessed *pre-pandemic* PPT and boredom ratings remained largely stable across the survey period. This stability suggests that the retrospective *pre-pandemic* ratings were of fairly high precision, i.e., showed little variation of the judgments within respondents over time, although we cannot finally evaluate the accuracy of the ratings, i.e., the congruence of the absolute level of the judgments with the actual *pre-pandemic* level. Hence, we are inclined to interpret the convergence of the assessed *pandemic* ratings with the retrospective *pre-pandemic* ratings as an “adaptation” to recalled *pre-pandemic* levels—rather than a “normalization” to the actual *pre-pandemic* levels.

## Conclusion

In the present study, we have demonstrated the adaptive nature of PPT and boredom during the Covid-19 pandemic using a longitudinal survey method. Even though the pandemic-related restrictions continued, people seemed to progressively adapt to these circumstances. This was indicated by their ratings of PPT and boredom, and further supported by their predictions for the duration of the pandemic. We suppose that respondents developed efficient strategies to cope with the social isolation during the pandemic, which might have directly influenced the level of boredom and the emotional state. We conclude that the perceived boredom and the general emotional state are important predictors of PPT under social distancing measures.

## Supplementary Information

Supplementary Information.

## Data Availability

The data of this study is available on the Open Science Framework: https://osf.io/jw9ak/?view_only=33f31b1f86144a26bd518aa103bf1721
