# Supplementary material for: Adapting to the pandemic: longitudinal effects of social restrictions on time perception and boredom during the Covid-19 pandemic in Germany
Source: Sci Rep. 2022 Feb 3;12:1863. doi: 10.1038/s41598-022-05495-2 (PMC8814006; doi:10.1038/s41598-022-05495-2)
Supplement: Supplementary file 1 — Supplementary Information. [file 41598_2022_5495_MOESM1_ESM.docx]

**Supplement**

Table S1.

Estimated fixed effects parameters of the model predicting pandemic boredom from the measurement point (MP). Shown are effect estimates (ß), standard errors (SE), degrees of freedom (df), t and p values. The difference coding compares the means of two consecutive measurement points.

|  | ***ß*** | ***SE*** | ***df*** | ***t*** | ***p*** |
| --- | --- | --- | --- | --- | --- |
| (Intercept) | 2.70 | 0.11 | 1128 | 23.88 | < .0001 |
| MP 2-1 | -0.41 | 0.13 | 1128 | -3.14 | .0017 |
| MP 3-2 | -0.17 | 0.13 | 1128 | -1.29 | .1959 |
| MP 4-3 | -0.35 | 0.13 | 1128 | -2.66 | .0080 |
| MP 5-4 | 0.00 | 0.13 | 1128 | -0.03 | .9767 |
| MP 6-5 | -0.04 | 0.13 | 1128 | -0.27 | .7870 |
| MP 7-6 | -0.02 | 0.13 | 1128 | -0.13 | .8943 |
| MP 8-7 | -0.28 | 0.13 | 1128 | -2.18 | .0297 |
| MP 9-8 | 0.16 | 0.13 | 1128 | 1.26 | .2071 |
| MP 10-9 | -0.11 | 0.13 | 1128 | -0.80 | .4232 |

Table S2.

Estimated fixed effects parameters of the model predicting pandemic PT from the measurement point (MP) and pandemic boredom. Shown are effect estimates (ß), standard errors (SE), degrees of freedom (df), t and p values. The difference coding compares the means of two consecutive measurement points.

|  | ***ß*** | ***SE*** | ***df*** | ***t*** | ***p*** |
| --- | --- | --- | --- | --- | --- |
| (Intercept) | 6.14 | 0.10 | 1127 | 62.86 | < .0001 |
| MP 2-1 | 0.10 | 0.11 | 1127 | 0.97 | .3344 |
| MP 3-2 | -0.03 | 0.11 | 1127 | -0.26 | .7932 |
| MP 4-3 | 0.01 | 0.11 | 1127 | 0.13 | .8981 |
| MP 5-4 | -0.13 | 0.11 | 1127 | -1.19 | .2360 |
| MP 6-5 | 0.22 | 0.11 | 1127 | 1.99 | .0463 |
| MP 7-6 | -0.07 | 0.11 | 1127 | -0.62 | .5368 |
| MP 8-7 | 0.02 | 0.11 | 1127 | 0.20 | .8449 |
| MP 9-8 | 0.08 | 0.11 | 1127 | 0.78 | .4378 |
| MP 10-9 | -0.05 | 0.11 | 1127 | -0.44 | .6631 |
| Boredom | -0.35 | 0.02 | 1127 | -15.20 | < .0001 |
